# Patient safety culture in a large teaching hospital in Riyadh: baseline assessment, comparative analysis and opportunities for improvement

**DOI:** 10.1186/1472-6963-14-122

**Published:** 2014-03-12

**Authors:** Fadi El-Jardali, Farheen Sheikh, Nereo A Garcia, Diana Jamal, Ayman Abdo

**Affiliations:** 1 Department of Health Management and Policy, Faculty of Health Sciences, American University of Beirut, Beirut, Lebanon; 2 Department of Clinical Epidemiology and Biostatistics, McMaster University, Canada, MML-417, 1280 Main St. West, Hamilton, Ontario L8S 4L6, Canada; 3 King Saud University, Riyadh, Kingdom of Saudi Arabia

## Abstract

**Background:**

In light of the immense attention given to patient safety, this paper details the findings of a baseline assessment of the patient safety culture in a large hospital in Riyadh and compares results with regional and international studies that utilized the Hospital Survey on Patient Safety Culture. This study also aims to explore the association between patient safety culture predictors and outcomes, considering respondent characteristics and facility size.

**Methods:**

This cross sectional study adopted a customized version of the HSOPSC and targeted hospital staff fitting sampling criteria (physicians, nurses, clinical and non-clinical staff, pharmacy and laboratory staff, dietary and radiology staff, supervisors, and hospital managers).

**Results:**

3000 questionnaires were sent and 2572 were returned (response rate of 85.7%). Areas of strength were Organizational Learning and Continuous Improvement and Teamwork within units whereas areas requiring improvement were hospital non-punitive response to error, staffing, and Communication Openness. The comparative analysis noted several areas requiring improvement when results on survey composites were compared with results from Lebanon, and the United States. Regression analysis showed associations between higher patient safety aggregate score and greater age (46 years and above), longer work experience, having a Baccalaureate degree, and being a physician or other health professional.

**Conclusions:**

Patient safety practices are crucial toward improving overall performance and quality of services in healthcare organizations. Much can be done in the sampled organizations and in the context of KSA in general to improve areas of weakness and further enhance areas of strength.

## Background

Patient safety has become a major priority to policymakers, healthcare providers and managers. Instigating a strong patient safety culture is pivotal for promoting this concept among healthcare professionals and sustaining this concept in healthcare organizations. Making patient safety culture a top priority is dependant on having a strong and positive patient safety culture [1]. Some components of a strong patient safety culture include open communication, teamwork, and acknowledged mutual dependency [2]. Assessing a healthcare organization’s patient safety culture is the first step for developing a strong and solid safety culture [3]. Reflecting that, many international accreditation organizations now require patient safety culture assessments to evaluate the perception of healthcare staff on issues such as teamwork, actions taken by management and leadership to support and promote patient safety, staffing issues, frequency of incident reporting, and other patient safety culture issues [4]. Such assessments allow healthcare organizations to obtain a clear view of areas requiring attention to strengthen their patient safety culture [5] and identify specific challenges relating to patient safety within hospital units [4]. Most importantly, healthcare organizations conducting such assessments can benchmark their results against similar surveys conducted within their country or on an international level [6]. A widely used tool for evaluating patient safety culture is the *Hospital Survey on Patient Safety Culture* (HSOPSC) [7]. The HSOPSC measures 12 patient safety culture composites representing several patient safety culture predictors. The HSOPSC also requires respondents to give their work area/unit a patient safety grade and to answer a question on the number of events reported in the past 12 months [7].

Evidence in the literature identifies several predictors for a strong and positive patient safety culture such as communication, information flow between and across units, common vision on the importance of patient safety, solid and constant commitment from management and leadership, and a non-punitive approach to incident and error reporting [8]. Despite the wealth of evidence published on patient safety culture in recent years, there is limited literature on this topic in the Arab world and the Kingdom of Saudi Arabia (KSA) in particular. A previous study conducted in 2009 in Riyadh identified organizational learning as the most positive aspect and non-punitive response to error as the weakest aspect of patient safety culture in public and private hospitals [9]. In 2010, a similar study utilizing the HSOPSC identified organizational learning and continuous improvement, teamwork within units, and feedback and communication about errors as areas of strength and event reporting, non-punitive response to error, staffing, and teamwork across hospital units as areas requiring improvement [10]. A study conducted in Turkey identified teamwork within units and overall perceptions of safety as areas of strength and frequency of event reporting and non-punitive response to error as areas requiring improvement [11]. The study highlighted infrequent levels event reporting was low and the majority of staff did not report or provide feedback about errors [11].

The realm of patient safety culture was also explored in Lebanon through surveying 6807 hospital employees in 68 hospitals. Using an Arabic version of the HSOPSC, the study identified the most critical issues related to patient safety culture and potential strategies to implement the patient safety accreditation standards. Areas of strength as reported by sampled respondents included teamwork within units, hospital management support for patient safety, and organizational learning and continuous improvement. However, areas of weakness included teamwork across hospital units, hospital handoffs and transitions, staffing, and non-punitive response to error [12]. Approximately 60% of respondents reported not completing any event reports in the past 12 months and over 70% gave their units an “Excellent/Very Good” patient safety grade. An aggregate score for all patient safety composites was regressed against respondent characteristics. A higher aggregate score was associated with longer years of experience, being nurses or pharmacists, interaction with patients, and working at an accredited hospital [12]. A follow up study was conducted to identify predictors and outcomes of patient safety culture. Better patient safety grades were associated with higher scores on supervisor/manager expectations, organizational learning and continuous improvement, teamwork within hospital units, feedback and communication about errors, staffing, and hospital management support for patient safety and hospital handoffs and transitions. Event reporting was found to be associated with higher scores on for feedback and communication about errors and lower scores on hospital handoffs and transition. Frequency of event reporting was associated with higher scores on organizational learning and continuous improvement, communication and openness, feedback and communication, non-punitive response to errors, hospital management support for patient safety, and teamwork across hospital units. Better overall perception about patient safety was associated with higher scores on supervisor/manager expectations and actions promoting safety, organizational learning and continuous improvement, teamwork within hospital units, non-punitive response to error, hospital management support for patient safety, and hospital handoffs and transitions [13].

### Objectives

The objective of this study was to conduct a baseline assessment of the patient safety culture in a large hospital in Riyadh, Kingdom of Saudi Arabia (the hospital is composed of two sites, one large with 800 beds and the other small with 104 beds) and compare results with regional and international studies that utilized the same tool. This study aimed at exploring the association between patient safety culture predictors and outcomes, taking into consideration respondent characteristics and facility size.

## Methods

### Design, setting and sampling

This cross sectional study adopted a customized version of the Hospital Survey on Patient Safety Culture (HSOPSC) developed by the Agency for Healthcare Research and Quality. The survey tool was translated to Arabic to account for employees who are not very comfortable with English. It should be noted that the Arabic version of the survey was adapted from a study conducted in Lebanon which utilized this translated version [12,13]. The survey was pilot tested with 20 employees who were not included in the final sample. Few modifications were done to the questionnaire as a result and they were mainly limited to adding more departments in the distribution list.

The hospital is a tertiary care university facility that has a total capacity of 904 beds with all major medical specialties and services. It receives referral patients from all over the country. The hospital is composed of two sites, Site A which is large (800 beds) and Site B which is small (104 beds).

The survey targeted selected hospital staff including physicians, nurses, clinical and non-clinical staff, pharmacy and laboratory staff, dietary and radiology staff, supervisors, and hospital managers. The two sites had a total of 5200 hospital employees of which 3000 fit the inclusion criteria. Data collection spanned several months and was available in electronic format (December 2011 to March 2012) and hard copies (February 2012 to March 2012). The first page of the survey included a consent form which included information about the study and definitions of terms used. Employees were not asked to sign on the consent form or any other page of the survey; they were asked to return the hard copies of the survey in sealed envlopes to one of 38 available collection boxes placed in designated Points of Contact (POC) assigned to different departments.

### Data management and analysis

Data collected using the hard copies of the survey were entered using MS Excel. Surveys filled electronically were saved onto a web-based application then exported for data analysis on SPSS. Data was analyzed using SPSS 19.0 at a significance level of 0.05. The HSOPSC is composed of 42 items that measure 12 composites. The HSOPSC included both positively and negatively worded items. Items were scored using a five-point scale reflecting the agreement rate on a five-point frequency scale (both including a neutral category). The percentage of positive responses for each item and composite was calculated; negatively worded items were reversed when computing percent positive response. For example, the responses “Strongly Agree/Agree” or “Most of the time/Always” are positive responses for positively worded items whereas for reverse worded items, disagreement indicates a positive response, so the responses “Strongly Disagree/Disagree” or “Never/Rarely” are considered. After counting percent positive responses per composite, this number is divided by the total number of responses for this composite to obtain a percent positive. Composite level scores were computed by summation of the items within the composite scales and dividing by the number of items with non-missing values. Cronbach’s alpha was used to measure the internal consistency of the 12 composites.

Two of the composites (frequency of events reported and overall perception of safety) are two of the four patient safety culture outcome variables [7]. The remaining two outcome variables are the patient safety grade and the number of events reported [7]. Pearson correlation was used to examine the association between frequency of frequency of events reported and overall perception of safety and the remaining 10 composites at the bi-variate level. Additionally, ANOVA f-test with multiple correction using Bonferroni was used to examine the association between patient safety grade and number of events reported across the two sites (Sites A and B). Cross-tables were constructed and chi-square test was used to examine the association between patient safety grade and number of events reported across the two sites (Sites A and B).

Calculating the item-level and the composite-level percent of positive and negative responses allowed for the identification of areas of strength (at least 75% of respondents answer positively) and areas with potential for improvement (less than 50% answer positively). As such, higher values reflect better scores. Univariate analysis was also conducted to summarize demographic characteristics of hospitals and respondents. Bi-variate analysis was conducted to derive potential variables to be included in the regression analyses. All tests were conducted at a significance level of 0.05.

The four outcome variables were regressed against the 10 composite scores, respondent’s position in the hospital, and hospital size. Four regression models were constructed, two adopted Generalized Estimating Equations (the two categorical outcome variables: number of events reported and patient safety grade) and the other two models followed a linear regression model (the two composites for frequency of events reported and overall perception of safety). In the latter models, the independent variables were entered as dummy variables. The two categorical outcomes were recoded into fewer categories for the purpose of this analysis. The outcome on patient safety grade was recoded into three categories “Poor or Failing,” “Acceptable,” and “Excellent/Good.” The outcome on number of events reported was recoded into “>5 events reported,” “1 to 5 events reported,” and “No events reported”.

Prior to data analysis, a data check was conducted to determine whether any of the respondents answered similarly to all questions (filled in the same value for all items). The survey was 3 pages long and as such, a total of 59 surveys for which respondents indicated the same value for 80% of the questions on the same page were dropped from the analysis. It was initially assumed that respondents would indicate the same value for the entire survey. When this assumption was assessed, only 18 cases were found.

Results on the survey composite were compared against those in three other countries, specifically, Lebanon, and the United States. A weighting exercise was conducted on SPSS to determine whether the difference in results from each country were significantly different. The percent positive responses for each of the composites were grouped in an MS Excel sheet and countries were compared collectively and in pairs. Given differences in types of health facilities, size of facilities, number of respondents, and other additional factors, this analysis was done for comparative purposes.

## Results

### General results

A total of 2,572 of the 3,000 questionnaires sent to the two sites were returned complete (2033 from Site A and 539 from Site B) yielding an overall response rate of 85.7%. Responses to the electronic format of the survey were 1524 (59.3%) whereas 1047 employees returned the hard copy of the survey (40.7%). The majority of sampled respondents were females (71.4%) with approximately half within the 30 to 45 age group (45.3%). A total of 43.6% held a Diploma while 38.9% had a Baccalaureate Degree (Table 1). Around a third of respondents indicated working in the Administration (27.1%) while 20.1% worked in Medical units, 19.6% in Surgical units and 21.2% in other units. Half the sampled respondents indicated being Registered Nurses (50.1%), 12.0% were Technicians, 6.1% were Attending/Staff Physicians, and 5.2% were Unit assistants, clerks or secretaries (Table 1). A third of respondents had 1 to 5 years of experience (30.5%) while 25% had 6 to 10 years of experience and 18.6% had less than 1 year of experience. The majority of respondents indicated that their work required direct contact with patients (76.1%). Approximately half the sampled respondents gave their hospital a Very Good patient safety grade (49%). Slightly over half the sampled respondents reported no events (52.7%), approximately a third (28.7%) reported 1 to 2 events, and 13% reported 3 to 5 events. It is worth noting that only 1.4% of respondents reported 21 or more events (Table 1).

**Table 1:** Socio-demographic and professional characteristics of respondents in addition to frequency of events and patient safety grade

**Characteristics**	**N**	**%**
**Gender**		
Male	728	28.6%
Female	1820	71.4%
**Age (years)**		
<30	854	33.7%
30–45	1148	45.3%
46–55	401	15.8%
≥55 years	133	5.2%
**Degree classification**		
Under high school	14	0.6%
High school level	62	2.5%
Diploma level	1082	43.6%
Baccalaureate degree	966	38.9%
Masters degree	124	5.0%
Doctorate degree	112	4.5%
Other	122	4.9%
**Work area/unit where respondents spend most of their work time**		
Many different hospital units/no specific unit	13	0.5%
Administration	697	27.1%
Medical	536	20.8%
Surgical	503	19.6%
Diagnostics	277	10.8%
Other	545	21.2%
**Respondents’ positions at the hospital**		
Administration/Management	92	3.6%
Attending/Staff physician	158	6.1%
Dietician	46	1.8%
Infection control practitioner/Coordinator/Nurse	13	0.5%
Patient care assistant/Hospital aide/Care partner	45	1.8%
Pharmacist	56	2.2%
Physical, occupational, speech therapist	52	2.0%
Physician assistant/Nurse practitioner	17	0.7%
Registered nurse	1287	50.1%
Resident physician/Physician in training	67	2.6%
Respiratory therapist	10	0.4%
Quality staff	23	0.9%
Unit assistant/Clerk/Secretary	133	5.2%
Technician (e.g., EKG, Lab, Radiology)	308	12.0%
Other, please specify:	264	10.3%
**Experience in current hospital (years)**		
Less than 1 year	463	18.6%
1 to 5 years	758	30.5%
6 to 10 years	622	25.0%
11 to 15 years	290	11.7%
16 to 20 years	136	5.5%
21 years or more	217	8.7%
**Experience in current work area (years)**		
Less than 1 year	436	17.3%
1 to 5 years	986	39.1%
6 to 10 years	528	20.9%
11 to 15 years	245	9.7%
16 to 20 years	159	6.3%
21 years or more	168	6.7%
**Job involves direct contact with patients**		
Yes	1956	76.1%
No	615	23.9%
**Patient safety grade**		
Excellent	507	20.6%
Very good	1207	49.0%
Acceptable	632	25.6%
Poor	100	4.1%
Failing	19	0.8%
**Number of events reported**		
No events	1275	52.7%
1 to 2 event reports	677	28.0%
3 to 5 event reports	315	13.0%
6 to 10 event reports	84	3.5%
11 to 20 event reports	36	1.5%
21 event reports or more	34	1.4%

### Determining Areas of strengths and areas requiring improvement according to patient safety culture composites results

**Figure 1:**
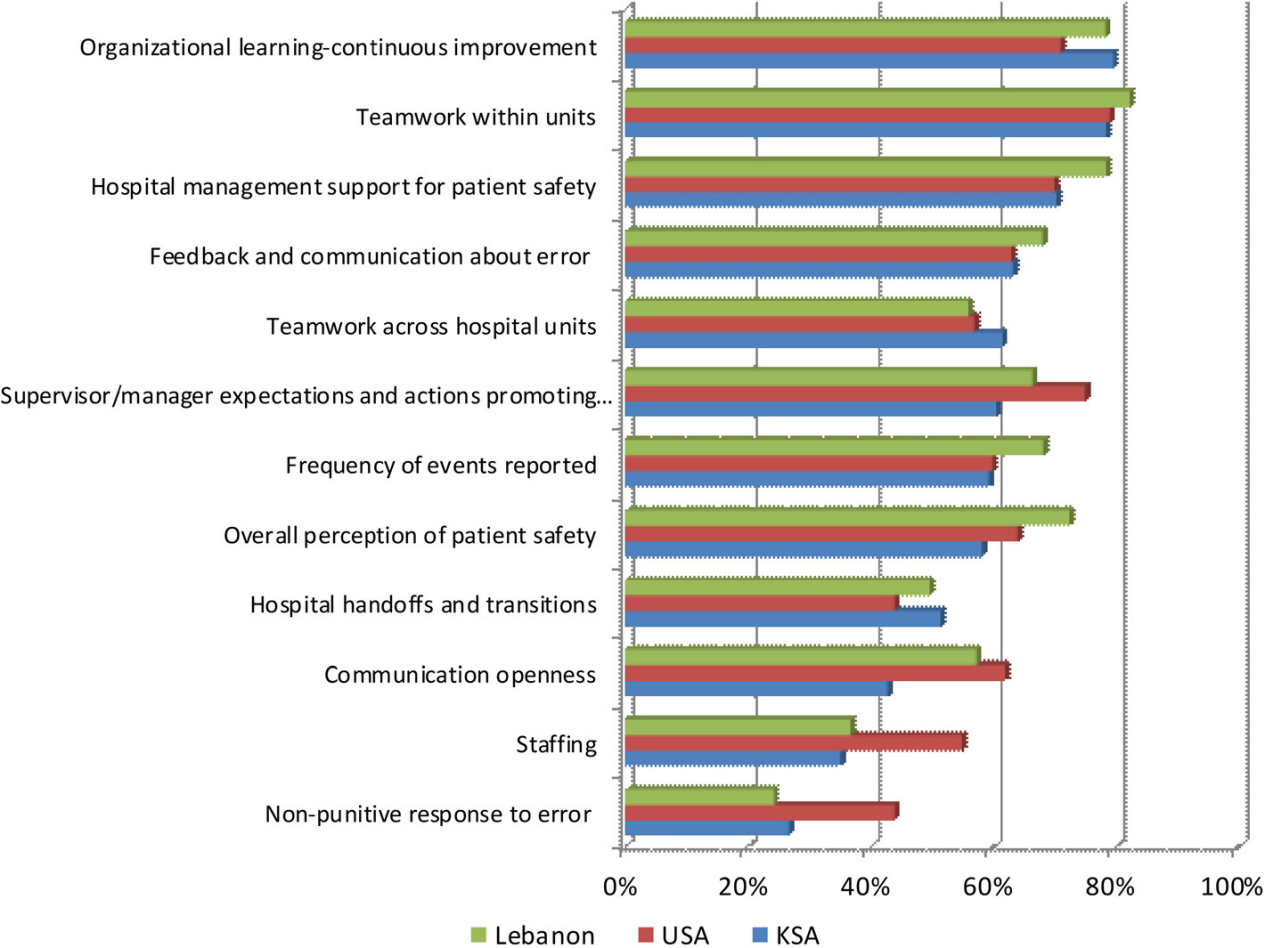
Composite-level average% positive response for KSA compared to that of Lebanon and USA.

The twelve dimensions were examined to determine areas of strength (those where percent positive rating exceeds 75%) and those requiring improvement (scoring below 50%). The dimensions with the highest positive score and are thus considered areas of strength were Organizational Learning and Continuous Improvement (79.6%), and Teamwork within units (78.5%). Dimensions scoring the lowest and as such can be considered areas requiring improvement were hospital non-punitive response to error (26.8%), staffing (35.1%), and Communication Openness (42.9%) (Figure 1).

Items considered areas of strength and other requiting improvement were then examined. One major area of strength was highlighted by the responses to the item on whether the hospital is actively doing things to improve patient safety to which percent positive response was 90%. Other areas of strength were revealed within the dimension on Teamwork within units whereby the item on whether staff support one another within a unit received 84.7% positive responses, whereas the item on the degree to which staff we work together as a team to get the work done when there is a lot of work to be done received 81.6% percent positive scores. Moreover, 80.7% of respondents responded positively when it came to whether staff members treat each other with respect within the unit (Table 2). Some of the areas requiring improvement pertained to the dimension on staffing whereby respondents reported that staff work longer hours than best for patient safety (19.3% positive) and 24.8% indicated that they try to do too much too quickly when work is in “crisis mode.” Other areas requiring improvement pertained to non-punitive response to error whereby 31.4% of staff felt like their mistakes were held against them, and 32.5% indicated feeling as though they were being written up when an event is reported (Table 2). Additional areas of strength and those requiring improvement are detailed in Table 2.

**Table 2:** Cronbach’s Alpha and distribution of positive responses and scores for survey composites and items

**Composites and survey items**	Average % positive response*	**Mean standard deviation**
**Overall perception of safety (Cronbach’s α = 0.214)**	**65.3**	**3.43 (0.59)**
It is just by chance that more serious mistakes do not happen around here (R)**	36.9	2.99 (1.16)
Patient safety is never sacrificed to get more work done	70.6	3.71 (1.07)
We have patient safety problems in this unit (R)	50.0	3.25 (1.15)
Our policies and procedures and systems are effective in preventing errors	75.3	3.76 (0.93)
**Supervisor/Manager expectations & actions promoting patient safety (Cronbach’s α = 0.436)**	**60.6**	**3.46 (0.65)**
My supervisor/manager says a good word when he/she sees a job done according to established patient safety procedures	71.8	3.69 (1.05)
My supervisor/manager seriously considers staff suggestions for improving patient safety	74.8	3.77 (0.97)
Whenever pressure builds up, my supervisor/manager wants us to work faster, even if it means taking shortcuts (R)	51.0	3.27 (1.09)
My supervisor/manager overlooks patient safety problems that happen over and over (R)	44.5	3.13 (1.18)
**Organizational learning and continuous improvement (Cronbach’s α = 0.704)**	**79.6**	**3.89 (0.69)**
We are actively doing things to improve patient safety	90.0	4.19 (0.82)
Mistake have led to positive changes here	69.5	3.63 (0.93)
After we make changes to improve patient safety, we evaluate their effectiveness	79.3	3.84 (0.87)
**Teamwork within units (Cronbach’s α = 0.814)**	**78.5**	**3.85 (0.75)**
Staff support one another in this unit	84.7	3.97 (0.88)
When a lot of work needs to be done quickly, we work together as a team to get the work done	81.6	3.93 (0.89)
In this unit, people treat each other with respect	80.7	3.92 (0.91)
When members of this unit get really busy, other members of the same unit help out	66.7	3.57 (1.08)
**Non-punitive response to error (Cronbach’s α = 0.643)**	**26.8**	**2.68 (0.81)**
Staff feel like their mistakes are held against them (R)	31.4	2.83 (1.09)
When an event is reported, it feels like the person is being written up, not the problem (R)	32.5	2.85 (1.09)
Staff worry that mistakes they make are kept in their personnel file (R)	16.5	2.37 (1.02)
**Staffing (Cronbach’s α = 0.238)**	**35.1**	**2.84 (0.62)**
We have enough staff to handle the workload	47.5	3.05 (1.20)
Staff in this unit work longer hours than is best for patient care (R)	19.3	2.41 (1.09)
We use agency/temporary staff than is best for patient care (R)	48.8	3.34 (1.11)
When the work is in “crisis mode” we try to do too much, too quickly (R)	24.8	2.58 (1.09)
**Hospital management support for patient safety (Cronbach’s α = 0.577)**	**70.4**	**3.69 (0.76)**
Hospital management provides a work climate that promotes patient safety	76.8	3.76 (0.96)
The actions of hospital management show that patient safety is a top priority	79.6	3.97 (0.96)
Hospital management seems interested in patient safety only after an adverse event happens (R)	54.6	3.32 (1.15)
**Teamwork across hospital units (Cronbach’s α = 0.643)**	**61.6**	**3.36 (0.79)**
There is good cooperation among hospital units that need to work together	65.5	3.58 (0.96)
Hospital units work well together to provide the best care for patients	77.1	3.90 (0.96)
Hospital units do not coordinate well with each other and this might affect patient care (R)	53.6	3.29 (1.12)
It is often not easy to work with staff from other hospital units (R)	50.1	3.30 (1.03)
**Hospital handoffs & transitions (Cronbach’s α = 0.770)**	**51.5**	**3.52 (0.71)**
Things “fall between the cracks”, i.e., things might go uncontrolled and get lost (ex: medical records, medical treatment, patient information and education, discharge criteria) when transferring patients from one unit to another (R)	43.5	3.20 (1.04)
Important patient care information is often lost during shift changes (R)	62.3	3.59 (1.02)
Problems often occur in the exchange of information across hospital units (R)	41.6	3.15 (0.99)
Shift changes are problematic for patients in this hospital (R)	58.5	3.51 (1.05)
**Communication openness (Cronbach’s α = 0.536)**	**42.9**	**3.25 (0.85)**
Staff will freely speak up if they see something that may negatively affect patient care	54.2	3.53 (1.16)
Staff feel free to question the decisions or actions of those with more authority	33.3	2.94 (1.23)
Staff are afraid to ask questions when something does not feel right (R)	41.1	3.30 (1.16)
**Feedback and communications about error (Cronbach’s α = 0.793)**	**63.3**	**3.73 (0.95)**
We are given feedback about changes put into place based on event reports	48.9	3.39 (1.13)
We are informed about errors that happen in this unit	68.9	3.85 (1.147)
In this unit, we discuss ways to prevent errors from happening again	72.0	3.94 (1.1)
**Frequency of events reported (Cronbach’s α = 0.892)**	**59.4**	**3.63 (1.16)**
When a mistake is made, but is caught and corrected affecting the patient, how often is this reported?	58.2	3.59 (1.29)
When a mistake is made, but has no potential to harm the patient, how often is this reported?	56.3	3.56 (1.27)
When a mistake is made that could harm the patient, but does not, how often is this reported?	63.7	3.74 (1.29)

*the composite-level percentage of positive responses was calculated using the following formula: (number of positive responses to the items in the composite/total number of responses to the items (positive, neutral, and negative) in the composite (excluding missing responses))*100.

**Negatively worded items that were reverse coded.

### Comparative against regional and international findings

Patient safety culture composite scores were compared to similar studies done in Lebanon, and the United States. The sampled hospital in Riyadh appeared to fare better on the dimension pertaining to Teamwork across hospital units, Hospital Handoffs and Transitions and Organizational learning and continuous improvement than hospitals in Lebanon [12], and the US [14]. Compared to Lebanon, the sampled hospital scores much lower on Hospital management support for patient safety, Feedback and communication about error, Supervisor Manager Expectations and Actions Promoting Patient Safety, Frequency of Events Reported, Overall Perception of Patient Safety, and Communication and Openness (Figure 1). When comparing findings against those of the study conducted in the US [14], the sampled hospital was found to need improvement on several composites including Non-punitive response to error, Staffing, Communication openness, Overall perception of patient safety, and Supervisor Manager Expectations and Actions Promoting Patient Safety (See Figure 1).

### Correlations between patient safety culture composites

Despite one exception, the 12 composites were found to be significantly but moderately correlated with varying degrees in the strength of these correlations. Within the composite on frequency of events reported, the strongest correlation was observed for feedback and communication about error (Pearson r = 0.413) while the weakest correlation was for that on supervisor manager expectations to promote patient safety (Pearson r = 0.081). It was interesting to observe a negative (albeit relatively weak) correlation between staffing and frequency of events reported (Pearson r = -0.087) (See Table 3).

**Table 3:** Correlations between patient safety culture composites

	**Frequency of events reported**		**Overall perception of safety**	
	**Pearson r**	**N**	**Pearson r**	**N**
Supervisor/Manager expectations and actions promoting safety	0.081*	1328	0.323*	1304
Organizational learning-continuous improvement	0.311*	1341	0.287*	1318
Teamwork within hospital units	0.186*	1349	0.297*	1326
Communication openness	0.168*	1342	0.184*	1311
Feedback and communication about errors	0.413*	1345	0.203*	1315
Non-punitive response to error	0.023	1323	0.176*	1304
Staffing	-0.087*	1328	0.222*	1310
Hospital management support for patient safety	0.265*	1344	0.352*	1317
Hospital handoffs and transitions	0.175*	1311	0.222*	1284
Teamwork across hospital units	0.209*	1321	0.276*	1292

*Correlation is significant at the 0.01 level (2-tailed).

As for the composite on overall perception of patient safety, the strongest correlation was that for hospital management support for patient safety (Pearson r = 0.352) while the weakest was that for non-punitive response to error (Pearson r = 0.176). It was also interesting to observe a weak correlation between communication and openness and overall perception of patient safety (See Table 3).

### Comparison of means between outcome variables and patient safety composites

Significantly different mean scores were observed for patient safety grade and all 10 patient safety culture composites with highest mean scores observed for respondents who indicated an Excellent/Very Good grade (See Table 4). The outcome of number of events reported was significantly associated with feedback and communication about error, hospital management support for patient safety, hospital handoffs and transitions, and teamwork across hospital units with the highest mean scores observed for respondents reporting 1 to 5 events (See Table 4).

**Table 4:** Comparison of means between patient safety grade and number of events reported with patient safety culture composite scores

	**Patient safety grade**				**Number of events reported**			
	**Poor or failing**		**Acceptable**	**Excellent/Very good**	**No event reports**		**1 to 5 event reports**	**>5 events reported**
	**Sig.**	**Mean (SD)**	**Mean (SD)**	**Mean (SD)**	**Sig.**	**Mean (SD)**	**Mean (SD)**	**Mean (SD)**
Supervisor/Manager expectations and actions promoting safety	a,b,c	2.94 (0.78)	3.29 (0.67)	3.60 (0.59)		3.51 (0.64)	3.49 (0.64)	3.53 (0.66)
Organizational learning-continuous improvement	a,b,c	2.95 (1.03)	3.80 (0.59)	4.10 (0.45)		3.96 (0.58)	4.04 (0.54)	3.96 (0.69)
Teamwork within hospital units	a,b,c	3.12 (1.05)	3.67 (0.67)	4.07 (0.53)		3.94 (0.61)	3.96 (0.61)	3.91 (0.93)
Communication openness	a,b,c	2.53 (1.08)	2.91 (0.81)	3.43 (0.80)		3.27 (0.84)	3.26 (0.86)	3.39 (0.88)
Feedback and communication about errors	a,b,c	2.68 (1.13)	3.55 (0.84)	4.08 (0.73)	a	3.81 (0.90)	4.01 (0.75)	3.94 (0.88)
Non-punitive response to error	c	2.56 (0.96)	2.43 (0.74)	2.75 (0.78)		2.65 (0.76)	2.65 (0.81)	2.81 (0.90)
Staffing	c	2.74 (0.58)	2.75 (0.55)	2.90 (0.63)		2.90 (0.61)	2.83 (0.62)	2.75 (0.63)
Hospital management support for patient safety	a,b,c	2.52 (0.70)	3.46 (0.68)	3.97 (0.60)	c	3.79 (0.73)	3.84 (0.67)	3.64 (0.74)
Hospital handoffs and transitions	a,b,c	3.03 (0.83)	3.19 (0.74)	3.56 (0.73)	c	3.41 (0.78)	3.52 (0.74)	3.31 (0.71)
Teamwork across hospital units	a,b,c	2.68 (0.68)	3.30 (0.64)	3.72 (0.64)	c	3.56 (0.72)	3.64 (0.64)	3.38 (0.72)

Patient Safety Grade.

a. Significant difference between “Poor or Failing” and “Acceptable”.

b. Significant difference between “Poor or Failing” and “Excellent/Very Good”.

c. Significant difference between “Acceptable” and “Excellent/Very Good”.

Number of Events Reported.

a. Significant difference between “No events reported” and “1 to 5 events reported”.

b. Significant difference between “No events reported” and ” > 5 events reported”.

c. Significant difference between ”1 to 5 events reported” and “ > 5 events reported”.

### Comparison of responses across the two facilities

Patient safety composites were significantly different across the two facilities of different size. Specifically, higher scores were observed in the small facility for the composites measuring overall perception of safety, supervisor manager expectations and actions to promote safety, teamwork within hospital units, communication and openness, non-punitive response to error, hospital management support for patient safety, and teamwork across hospital units (See Table 5).

**Table 5:** Comparing responses across the two facilities

	**Large**		**Small**		
	**N**	**Mean (SD)**	**N**	**Mean (SD)**	P-Value*
Frequency of event reporting	1054	3.84 (1.11)	321	3.71 (1.12)	0.067
Overall perceptions of safety	1032	3.42 (0.58)	316	3.56 (0.63)	***<0.001***
Supervisor/manager expectations and actions promoting safety	1043	3.48 (0.64)	323	3.59 (0.62)	***0.007***
Organizational learning-continuous improvement	1051	3.99 (0.59)	325	4.06 (0.49)	0.275
Teamwork within hospital units	1066	3.91 (0.66)	323	4.07 (0.53)	***<0.001***
Communication openness	1051	3.23 (0.87)	325	3.43 (0.79)	***<0.001***
Feedback and communication about errors	1054	3.92 (0.82)	325	3.86 (0.90)	0.298
Non-punitive response to error	1040	2.64 (0.80)	321	2.74 (0.77)	***0.046***
Staffing	1039	2.86 (0.63)	322	2.84 (0.61)	0.691
Hospital management support for patient safety	1064	3.75 (0.71)	320	3.96 (0.67)	***<0.001***
Hospital handoffs and transitions	1030	3.43 (0.77)	317	3.52 (0.74)	0.071
Teamwork across hospital units	1038	3.54 (0.69)	320	3.73 (0.67)	***<0.001***
		**N (%)**		**N (%)**	**P-Value**
Patient safety grade					
Poor or failing		38 (3.6%)		7 (2.1%)	***0.001***
Acceptable		277 (26.3%)		57 (17.4%)	
Excellent/Very good		740 (70.1%)		263 (80.4%)	
Number of events reported					
No event reports		505 (46.2%)		183 (54.5%)	***0.008***
1 to 5 event reports		508 (46.5%)		140 (41.7%)	
>5 events reported		80 (7.3%)		13 (3.9%)	

*Bold and italicized font is to refer to statistically significant p-values.

### Generalized estimating equations for the patient safety composite scores and respondent and hospital characteristics against the patient safety grade and the number of events reported

#### ***Patient safety grade***

As detailed in Table 6, a one unit increase on most patient safety culture composites increased odds of reporting better patient safety grades. In fact, patient safety grades increased by 2.80 (95% CI = 2.55 - 3.08) for every unit increase in Hospital Management Support for Patient Safety, 1.91 (95% CI = 1.63 - 2.25) for every unit increase in Organizational learning and Continuous Improvement and by 1.44 (95% CI = 1.33 - 1.56) for every unit increase in Feedback and Communications About Error. The only composite that was not found to be significantly associated with patient safety grade was that measuring Hospital Handoffs & Transitions.

**Table 6:** Generalized estimating equations

	**Patient safety grade**		**Number of events reported**	
	**OR (95% CI)**	P-value*	**OR (95% CI)**	P-value*
Patient safety culture composites				
Supervisor/Manager expectations & actions promoting patient safety	1.06 (1.05 - 1.08)	***<0.001***	0.90 (0.71 - 1.16)	0.430
Organizational learning and continuous improvement	1.91 (1.63 - 2.25)	***<0.001***	0.89 (0.81 - 0.98)	***0.018***
Teamwork within units	1.34 (1.14 - 1.59)	***0.001***	0.88 (0.84 - 0.92)	***0.000***
Communication openness	1.25 (1.20 - 1.31)	***<0.001***	1.05 (0.82 - 1.34)	0.710
Feedback and communications about error	1.44 (1.33 - 1.56)	***<0.001***	1.50 (1.49 - 1.51)	***<0.001***
Non-punitive response to error	1.13 (1.07 - 1.20)	***<0.001***	1.08 (1.04 - 1.13)	***<0.001***
Staffing	1.30 (1.28 - 1.32)	***<0.001***	0.76 (0.70 - 0.84)	***<0.001***
Hospital management support for patient safety	2.80 (2.55 - 3.08)	***<0.001***	0.83 (0.71 - 0.97)	***0.021***
Hospital handoffs & transitions	1.09 (0.94 - 1.26)	0.253	1.05 (0.95 - 1.16)	0.355
Teamwork across hospital units	1.13 (1.09 - 1.17)	***<0.001***	0.94 (0.83 - 1.06)	0.328
Gender				
Male	1		1	
Female	0.50 (0.39 - 0.65)	***<0.001***	0.75 (0.62 - 0.91)	***0.003***
Age				
Less than 30 years of age	1		1	
Between 30 and 45	0.91 (0.89 - 0.93)	***<0.001***	1.43 (0.96 - 2.13)	0.076
Between 46 and 55	0.74 (0.48 - 1.16)	0.188	1.23 (0.68 - 2.22)	0.488
Aged above 55	0.66 (0.49 - 0.89)	***0.006***	1.11 (0.42 - 2.98)	0.830
Experience at the hospital				
Less than 1 year	1		1	
1 to 5 years	1.01 (0.75 - 1.36)	0.928	0.42 (0.37 - 0.48)	0.928
6 to 10 years	1.13 (1.09 - 1.17)	***<0.001***	0.45 (0.37 - 0.54)	***<0.001***
11 to 15 years	1.13 (1.11 - 1.15)	***<0.001***	0.28 (0.26 - 0.31)	***<0.001***
16 to 20 years	1.31 (1.05 - 1.64)	***0.018***	0.20 (0.12 - 0.32)	0.018
More or equal to 21 years	0.88 (0.56 - 1.38)	0.578	0.27 (0.16 - 0.48)	0.578
Highest degree				
High school level or below	1		1	
Diploma level	0.29 (0.24 - 0.35)	***<0.001***	18.88 (14.74 - 24.19)	***<0.001***
Baccalaureate degree	0.23 (0.22 - 0.25)	***<0.001***	26.13 (24.34 - 28.05)	***<0.001***
Masters degree	0.13 (0.09 - 0.17)	***<0.001***	47.61 (30.17 - 75.04)	***<0.001***
Doctorate degree	0.21 (0.09 - 0.50)	***<0.001***	56.09 (7.55 - 416.96)	***<0.001***
Other	0.24 (0.16 - 0.37)	***<0.001***	9.57 (7.86 - 11.66)	***<0.001***
Position at the hospital				
Administration/Management	1		1	
Attending/Staff physician	0.67 (0.57 - 0.79)	***<0.001***	0.37 (0.22 - 0.62)	***<0.001***
Dietician	0.09 (0.02 - 0.35)	***0.001***	0.74 (0.43 - 1.27)	0.273
Infection control practitioner/Coordinator/Nurse	0.22 (0.08 - 0.64)	***0.006***	0.91 (0.48 - 1.73)	0.781
Patient care assistant/Hospital Aide/Care partner	0.85 (0.30 - 2.35)	0.748		
Pharmacist	0.37 (0.06 - 2.16)	0.270	1.32 (1.06 - 1.65)	***0.015***
Physical, occupational, speech therapist	0.32 (0.08 - 1.19)	0.088	0.15 (0.12 - 0.18)	***<0.001***
Physician assistant/Nurse practitioner			0.68 (0.68 - 0.69)	***<0.001***
Registered nurse	0.35 (0.13 - 0.93)	***0.034***	0.86 (0.79 - 0.94)	***0.001***
Resident physician/Physician in training	0.29 (0.12 - 0.75)	***0.010***	1.14 (0.48 - 2.71)	0.770
Respiratory therapist	0.13 (0.06 - 0.27)	***<0.001***	1.58 (1.3 - 1.91)	***<0.001***
Quality staff	0.15 (0.03 - 0.65)	***0.011***	1.23 (0.49 - 3.06)	0.656
Unit assistant/Clerk/Secretary	0.32 (0.20 - 0.51)	***<0.001***	0.17 (0.13 - 0.21)	***<0.001***
Technician (e.g., EKG, Lab, Radiology)	0.95 (0.62 - 1.45)	0.803	0.32 (0.14 - 0.75)	***0.009***
Other	0.48 (0.11 - 2.06)	0.324	0.26 (0.11 - 0.59)	***0.001***
Interaction with patients				
No	1.08 (0.79 - 1.46)	0.633	1.18 (0.97 - 1.43)	0.102
Yes	1		1	
Hospital size				
Small	1.58 (1.25 - 1.99)	***<0.001***	0.62 (0.60 - 0.65)	***<0.001***
Large	1		1	
N	1005		1029	

*Bold and italicized font is to refer to statistically significant p-values.

Female respondents had 0.50 lower odds (95% CI = 0.39 - 0.65) of reporting better patient safety grades. Respondents aged between 30 and 45 and those above 55 also had lower odds of reporting better patient safety grades (OR = 0.91, 95% CI = 0.89 - 0.93 and OR = 0.66, 95% CI = 0.49 - 0.89 respectively). Experience was associated with higher patient safety grades whereby respondents who had 6 to 20 years of experience had greater odds of reporting higher patient safety grades (See Table 6). Degrees were negatively associated with patient safety grades whereby increasing educational attainment was associated with lower patient safety grades (See Table 6). As for respondent positions, attending physicians, dieticians, registered nurses, resident physicians, respiratory therapist, quality staff and unit assistants and clerks had lower odds of reporting higher patient safety grades. Respondents working in the small site of the hospital had significantly higher odds of reporting better patient safety grades (OR = 1.58, 95% CI = 1.25 - 1.99) (See Table 6).

#### ***Number of events reported***

A one unit increase in some composites was found to increase number of events whereby an increase in some others had the opposite effect. An increase in Feedback and Communication about Error, and non-punitive response to error led to higher odds of reporting higher number of events. Composites that were associated with lower odds of reporting higher number of events were: Organizational Learning and Continuous Improvement, Teamwork within Units, Staffing, and Hospital Management Support for Patient Safety (See Table 6).

Female respondents had 0.75 lower odds (95% CI = 0.62 – 0.91) of reporting higher number of events. While respondent age was not significantly associated with number of events, a work experience ranging between 6 to 15 years was associated with lower odds of reporting higher number of events. Increasing levels of education was also associated with much greater odds of reporting higher number of events. With regard to positions, all of Attending/Staff Physician; Physical, occupational speech therapists; physician assistant/nurse practitioners; unit assistant/clerks; technicians; and others had lower odds of reporting higher number of events. However, pharmacists and respiratory therapists had greater odds of reporting higher number of events. Working in the smaller hospital site was also associated with reporting lower number of events (OR = 0.62, 95%CI = 0.60 - 0.65) (See Table 6).

### Linear regression for the patient safety composite scores and respondent and hospital characteristics against the frequency of events reported and the overall perception of safety

#### ***Frequency of events reported***

Linear regression analysis showed that a one unit increase in the score on organizational learning and continuous improvement increased the frequency of events reported by 0.24 (P-Value = 0.002) whereas an increase in Feedback and Communications About Error increased frequency of events reported by 0.43 (p-value < 0.001). However, a one unit increase in Staffing and Teamwork across units decreased frequency of events by -0.17 (p-value = 0.002) and -0.15 (p-value = 0.031) respectively (See Table 7).

**Table 7:** Linear regression model

	**Frequency of events reported**		**Perception of patient safety**	
	**Beta (Standard error)**	P-value*	**Beta (Standard error)**	P-value*
Patient safety culture composites				
Supervisor/Manager expectations & actions promoting patient safety	-0.08 (0.06)	0.178	0.09 (0.03)	***0.008***
Organizational learning and continuous improvement	0.24 (0.08)	***0.002***	0.20 (0.04)	***<0.001***
Teamwork within units	0.04 (0.07)	0.506	0.09 (0.03)	***0.007***
Communication openness	-0.04 (0.05)	0.440	-0.06 (0.03)	***0.013***
Feedback and communications about error	0.43 (0.06)	***<0.001***	0.002 (0.03)	0.955
Non-punitive response to error	-0.02 (0.05)	0.715	0.006 (0.02)	0.817
Staffing	-0.17 (0.05)	***0.002***	0.13 (0.03)	***<0.001***
Hospital management support for patient safety	0.11 (0.06)	0.066	0.17 (0.03)	***<0.001***
Hospital handoffs & transitions	0.06 (0.05)	0.237	0.08 (0.03)	***0.004***
Teamwork across hospital units	-0.15 (0.07)	***0.031***	0.006 (0.04)	0.868
Gender				
Male	0.05 (0.10)	0.650	0.02 (0.05)	0.686
Female	0		0	
Age				
Less than 30 years of age	0		0	
Between 30 and 45	-0.09 (0.09)	0.337	-0.10 (0.05)	***0.036***
Between 46 and 55	0.08 (0.15)	0.572	-0.15 (0.08)	***0.046***
Aged above 55	0.20 (0.20)	0.317	-0.21 (0.10)	***0.046***
Experience at the hospital				
Less than 1 year	0		0	
1 to 5 years	0.03 (0.10)	0.791	-0.11 (0.05)	***0.047***
6 to 10 years	-0.02 (0.13)	0.867	-0.06 (0.07)	0.373
11 to 15 years	-0.01 (0.16)	0.927	-0.04 (0.08)	0.633
16 to 20 years	0.12 (0.18)	0.510	-0.004 (0.09)	0.962
More or equal to 21 years	-0.07 (0.20)	0.741	-0.04 (0.10)	0.649
Highest degree				
High school level or below	0		0	
Diploma level	-0.19 (0.31)	0.528	0.06 (0.15)	0.691
Baccalaureate degree	-0.23 (0.31)	0.446	0.08 (0.15)	0.587
Masters degree	-0.01 (0.34)	0.966	-0.10 (0.17)	0.561
Doctorate degree	0.12 (0.40)	0.758	-0.07 (0.20)	0.711
Other	-0.54 (0.36)	0.134	0.05 (0.18)	0.769
Position at the hospital				
Administration/Management	0.12 (0.23)	0.599	-0.05 (0.12)	0.701
Attending/Staff physician	-0.26 (0.28)	0.337	0.05 (0.14)	0.736
Dietician	-0.22 (0.38)	0.565	-0.33 (0.20)	0.102
Infection control practitioner/Coordinator/Nurse	0.21 (0.33)	0.53	-0.05 (0.17)	0.791
Patient care assistant/Hospital aide/Care partner	0.58 (0.29)	0.045	-0.11 (0.15)	0.479
Pharmacist	0.19 (0.29)	0.511	0.11 (0.16)	0.477
Physical, occupational, speech therapist	-0.22 (0.26)	0.393	-0.29 (0.13)	***0.029***
Physician assistant/Nurse practitioner	-0.05 (0.48)	0.925	-0.31 (0.25)	0.219
Registered nurse	0.29 (0.15)	0.06	-0.32 (0.07)	***<0.001***
Resident physician/Physician in training	-0.71 (0.30)	0.019	-0.13 (0.16)	0.408
Respiratory therapist	-0.19 (0.62)	0.759	-0.59 (0.33)	0.072
Quality staff	-0.18 (0.39)	0.638	-0.19 (0.19)	0.324
Unit assistant/Clerk/Secretary	0.24 (0.23)	0.298	-0.29 (0.12)	***0.019***
Technician (e.g., EKG, Lab, Radiology)	0.01 (0.17)	0.934	-0.15 (0.09)	0.104
Other	0		0	
Interaction with patients				
No	0		0	
Yes	0.06 (0.11)	0.567	0.004 (0.06)	0.939
Hospital size				
Small	0		0	
Large	0.09 (0.08)	0.268	-0.05 (0.04)	0.273
N	1008		1001	

*Bold and italicized font is to refer to statistically significant p-values.

#### ***Overall perception of safety***

Perception of patient safety improved by 0.09 (P-Value = 0.008) for a one unit increase in the score on supervisor/manager expectations and actions promoting safety, by 0.20 (P-Value < 0.001) for every unit increase in the score on organizational learning and continuous improvement, and by 0.09 (P-Value < 0.001) for a one unit increase in the score on teamwork within units. A one unit increase in the composites on Staffing, Hospital Management Support for Patient Safety, Hospital Handoffs & Transitions were also found to increase overall perception of patient safety by 0.13 (p-value < 0.0010, 0.17 (p-value <0.001) and 0.08 (p-value = 0.004) respectively. A one unit increase in Communication and Openness was associated with -0.06 lower overall perception of patient safety (p-value = 0.013) (See Table 7).

Longer years of experience were associated with progressively lower overall perception of patient safety as observed in Table 4. Moreover, respondents who had 1 to 5 years of experience had -0.11 (p-value = 0.047) lower perception of patient safety. Physical, Occupational, Speech Therapists, Registered Nurses and Unit Assistant/Clerk/Secretary all had lower overall perception of patient safety (Table 7).

## Discussion

This is one of the few large scale studies assessing patient safety culture in one organization in KSA. Findings were able to identify areas of strength (Organizational Learning and Continuous Improvement and Teamwork within units) and weaknesses (Non-punitive response to error, staffing, and Communication Openness). Study findings showed many areas for improvement, particularly in comparison to other countries in the region and around the world. Results on some composites such as non-punitive response to error, staffing, and communication and openness are lower than countries in the region and the US. However, patient safety culture initiatives are novel in the region since the topic only started garnering attention in the past five years. On the other hand, patient safety has been an area of debate and attention in the US since the publication of the Institute of Medicine (IOM) report in 2001 and as a result, it is natural to find better results on some areas compared to those in KSA and even Lebanon.

Findings at the bi-variate and multi-variate level point to several areas of improvement within the composites that can increase patient safety grade, number of events reported, frequency of events reported and perception of patient safety. It appeared that higher composites on communication and openness, feedback and communication about error and non-punitive response to error were associated with lower number of events reported. Moreover, higher scores on staging and teamwork across hospital units were associated with lower frequency of events reported and higher scores on communication and openness were associated with lower perception of patient safety. These finding can be linked to the responses to the question on frequency of event reporting imply a fear of reporting, and this can be linked to some respondents’ belief that mistakes were held against them when they report an incident. Fear of reporting can present an impediment to a positive patient safety culture. Reasons for not reporting errors as detailed in literature include fear, humiliation, and the presence of a punitive response to error [15]. There is a need to encourage health professionals, particularly nurses who comprised around half the sampled respondents, to report events given its positive impact in improving patient safety. In fact, evidence in the literature defines three major components to a positive patient safety culture, mainly a just culture, a reporting culture, and a learning culture [2]. A non-punitive environment where hospital employees feel free and confident in reporting events without fear of blame is essential for better event reporting and consequently a safer hospital [5]. Non-punitive response to error was also low in Lebanon where it scored lowest among all dimensions [12].

Staffing also appeared to be a challenge to respondents in this study, as they specifically indicated working longer hours than is best for patient safety, and that they try to do too much too quickly in crisis mode. This finding is critical given the large body of evidence linking the availability of health care providers to population health outcomes [16]. Organizations with an insufficient number of employees have suffered major patient related catastrophes [8]. In cases where the number of employees is lower than optimum to provide patient care, most staff are overworked, burned out, suffer from stress and sleeplessness which may cause lapses in performance which could affect quality and patient outcomes [8,17].

Despite the findings above, many areas of strength were also identified including actions taken by the hospital to improve patient safety, degree of staff support within a unit, working together as a team. Findings in Lebanon linked higher scores on teamwork across hospital units to frequency of events reported. Higher scores on hospital handoffs and transitions were linked to greater likelihood of better perception of safety and also greater likelihood of reporting a higher patient safety grade [12].

One interesting observation is that related to the impact of small hospital size on patient safety culture outcomes. The study noted a significant association between small facility size and higher patient safety grade and number of events reported. Small hospital size has been found to be associated with higher overall average percent positive response on the patient safety culture composites and a higher percentage of respondents giving their work areas a patient safety grade of “Excellent” or “Very Good” [18]. On a related note, a study in Lebanon found a link between hospital size and better quality results in a study on impact of accreditation on quality of care [19]. Size is particularly important to consider given that evidence from the literature documenting its effect on managerial practices in healthcare organizations. For instance, large organizations find it easier to comply with accreditation requirements (such as assessment of patient safety culture) and derive greater value and benefit compared to smaller organizations given the lower financial burden in comparison to their overall budgets [20]. However, the edge that smaller organizations possess is their homogenous culture and shared values [21]. Larger organizations are more hierarchical and bureaucratic making implementation of quality initiatives challenging [22]; this also affects employees’ attachment to these organizations and consequently their performance [21]. Evidence from international literature link small hospital size (<100 beds) to increased formal organizational leadership in relation to patient safety events which in effect lead to better patient safety behaviors at the organizational level. This is due to the fact that in small hospitals (where the economic burden of safety programs may be large), formal leadership is closer to the front lines and has greater impact than in larger hospitals [23].

Some strengths and limitations to this study should be acknowledged. One of the strengths of this study is its use of the HSOPSC which is the most commonly used tool to assess the culture of safety in hospitals. This study also utilized the Arabic version of the survey which was translated and validated in in another Arab country [12,13]. Despite the fact that most employees in the sampled organizations are expatriates and should thus be fluent in English, Arabic remains the native language that most employees feel more comfortable with. As such, providing employees with an Arabic version of the survey may have allowed them to better understand and respond to the specific items of the survey. It should be acknowledged that while this study targeted a major health care organization in Riyadh and was able to obtain a large sample size representing the majority of staff, results should be interpreted with caution and not be generalized. However, it does offer insight into the current status of patient safety culture and was able to build on and validate the findings of previous research, particularly after major work has been conducted on accreditation in healthcare organizations in KSA including the facilities in this specific study.

Some may consider the low Cronbach’s alpha values a limitation (values ranged between 0.214 and 0.892). The HSOPSC user’s guide indicates that a value equal to or greater than 0.6 is acceptable [7] whereas Bowling [24] sets 0.5 or above as an acceptable value. Still, evidence shows that lower values of Cronbach’s alpha are expected when using psychological constructs due to the diversity of the items that are being measured [25]. A study in Turkey also reported values as low as 0.4 [11]. In Lebanon, Cronbach’s alpha values were as low as 0.451 and were attributed to the wide range of respondents [12]. Exploring the Cronbach’s alpha across respondent’s positions in this study also showed variation which may indicate that respondents in specific positions may be more (or less) aware about specific areas of patient safety culture challenges. This finding may require additional analysis to understand how different positions within the hospital may affect performance and consequently quality of care.

There are limited opportunities for countries to compare their performance against countries in the same region or around the world. This is the first study to compare such results in the region and the results presented in the comparative analysis component detail areas where the findings in KSA are similar and different than Lebanon, and USA. The comparative analysis required obtaining detailed data from each country pertaining to percent positive responses and sample size. Detailed results were available for sample size for each of the composites for both Lebanon and KSA but not for USA. In the latter cases, the total number of cases was used instead which was 567,703 for USA. This may have affected the results of the comparative analysis. Despite these limitations, the significant differences with Lebanon and USA provide a number of insights for the hospital in Riyadh on areas of strength and areas requiring some additional improvement.

## Conclusion

Investing in practices that strengthen patient safety is crucial if hospitals are to improve overall performance and quality of services. There is significant work to be done in the sampled organizations and in the context of the region in general to improve patient safety practices and culture. Patient safety should be integrated into educational programs for health professionals and embedded within the foundation of organizational structures. Health professionals needed not only continuing education but organizational support in the form of policies, governance and reporting structures. There is a need to give priority to patient safety culture assessments in health organizations and more importantly to make changes based on the results of such assessments. Regular assessment and reporting of patient safety culture is also required by some national and international accreditation programs. Such assessments can indicate areas requiring improvement and as such help devise targeted efforts that focus on investing in and improving patient safety and overall performance. It should be noted that assessing the patient safety culture is only the first step in a long process for identifying areas for improvement that hospitals can address to avert critical patient outcomes. Longitudinal research based on regular assessment of patient safety culture is needed to determine whether tangible improvements resulted from implemented quality improvement plans and affected the culture of safety or had an impact on clinical outcomes.

### Ethical considerations

The protocol for the original study was approved by the American University of Beirut Internal Review Board (protocol number FHS.FE.03). No additional local ethical review was required as advised by hospital administration. The study adhered to all ethical considerations pertaining to confidentiality of the responses provided by employees and informed consent form was provided and anonymity of responses was ensured. The facility provided an anonymous drop box to return the questionnaire at clearly designated points. The survey was also available in an online format and adhered to all ethical standards.

## Abbreviations

HSOPSC: Hospital survey on patient safety culture; IOM: Institute of medicine; KSA: Kingdom of Saudi Arabia; OR: Odds ratio; POC: Points of contact.

## Competing interests

The authors declare that they have no competing interests.

## Authors’ contributions

FE contributed to the conception, study design, tool development, data collection, as well as data analysis and interpretation of results in addition to development of the manuscript. FS and NAG contributed to data collection and coordination in addition to manuscript review. DJ also contributed to data analysis and manuscript write-up and review. AA contributed to manuscript write-up and review of the final manuscript. All authors read and approved the final manuscript.

## Pre-publication history

The pre-publication history for this paper can be accessed here:

http://www.biomedcentral.com/1472-6963/14/122/prepub
